# The Management of an Unusual Combination of Anomalies Following Primary Tooth Trauma: A Case Report

**DOI:** 10.7759/cureus.61402

**Published:** 2024-05-31

**Authors:** Viswanathan Revathy, Dhanraj Kalaivanan, Lalitha S Jairam, Sumaiyya Saleem, Madhuram Krishnamoorthy

**Affiliations:** 1 Pediatric Dentistry, Tamil Nadu Government Dental College, Chennai, IND; 2 Pediatric Dentistry, Sathyabama Dental College and Hospital, Chennai, IND; 3 Pediatric Dentistry, Faculty of Dental Sciences, M. S. Ramaiah University of Applied Sciences, Bengaluru, IND; 4 Endodontics, Karpaga Vinayaga Institute of Dental Sciences, Chengalpet, IND

**Keywords:** maxillary, permanent tooth, cbct, prognosis, tooth luxation

## Abstract

The prognosis of luxation injuries of primary teeth is often unpredictable. A two-year-old girl presented with a displaced left primary maxillary anterior tooth diagnosed as severe intrusive luxation. On radiographic examination, it was noticed to be impinging on the underlying permanent tooth bud. The tooth was extracted, and periodic follow-ups were emphasized until the successor tooth erupted. However, after eight years, the patient followed up with a chief complaint of an unerupted permanent maxillary anterior tooth. Cone-beam computed tomography (CBCT) revealed a combination of anomalies in the crown and root of the maxillary anterior tooth. After a wait-and-observe regime for six months, the incisal third of the crown erupted. At eight years and eight months, the tooth developed a periapical abscess which was treated endodontically and restored. At nine years and three months, the tooth is asymptomatic till date. The present case report highlights the consequences of intrusive luxation injuries to the primary teeth and the need for a multidisciplinary team approach in the management of complications of dental trauma along with meticulous long-term follow-ups.

## Introduction

The primary teeth are highly liable to luxation (displacement) injuries constituting 21%-81% of all traumatic dental injuries (TDI) [1]. Intrusive luxation constitutes 8%-22% of all luxation injuries of the primary anterior teeth, with varied prevalences up to 54% [2]. Intrusive luxation results in axial displacement of the tooth into the alveolar bone [3]. The displacement might cause damage to the periodontal structures and pulp, resulting in crushing and rupture of the fibers of the periodontal ligament and vascular supply, with rupture of the neurovascular supply to the pulp. It may also cause a fracture of the alveolar bone [3].

Traumatic injuries to the primary teeth can cause various developmental problems in the permanent teeth. The level of permanent tooth damage is governed by various parameters including the developmental age of the tooth germ, the age of the child during trauma, the magnitude and direction of the trauma, and its proximity of primary tooth roots to the developing tooth bud. The prevalence of detecting an abnormal permanent tooth as a result of trauma to its predecessor has been shown to range between 12% and 74% [4].

Following an invasive injury, the developing permanent tooth is vulnerable to damage due to its proximity to the root of the primary tooth [5]. Intrusive luxation injuries have shown detrimental influences on the eruption and/or development of permanent successors, ranging from a minor disruption in the mineralization of enamel to the complete sequestration of the tooth germ [6]. The most severe implications of TDI in the young, immature successional tooth occur when the kid is younger than two years old. The above outcome might be explained by the surrounding bone being less calcified which does not protect the follicle as well as it does in older children and this young age corresponds with the early stages of odontogenesis [7].

Crown dilacerations and malformations are uncommon sequelae, accounting for just 3% of all permanent dental abnormalities [8,9]. Disturbance to the growing root causes root duplication or partial to total root dilaceration [4]. In the present case report, the authors discuss a unique case report of intrusive luxation in a two-year-old child resulting in a trio of reverberations in a single permanent tooth such as crown malformation, dilacerations of the root and root multiplication at the age of eight years and its interdisciplinary management.

## Case presentation

A two-year-old girl along with her parents reported to the Department of Pediatric and Preventive Dentistry, Tamilnadu Government Dental College, Chennai with the chief complaint of upwardly placed upper front tooth due to self-fall the day before from a walker. On further analysis, the child did not have any previous history of trauma which was further confirmed by her previous photographs taken by the parents. The child was otherwise physically normal with no contributory medical history. Her vaccination status was up to date as per schedule. History revealed that it was the child’s first dental visit. The family history was non-contributory.

Clinical examination and diagnosis

**Figure 1:**
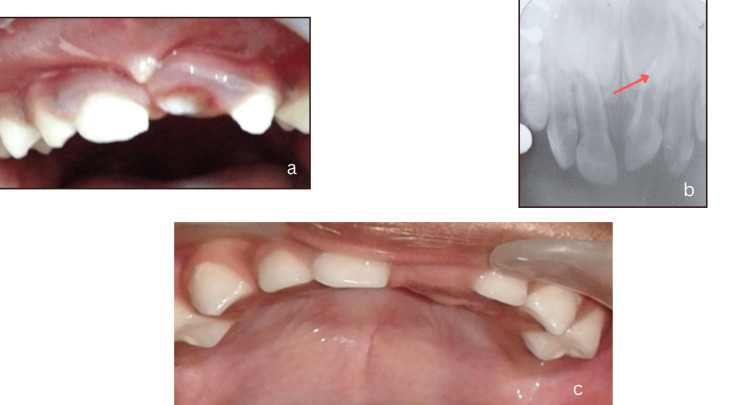
(a) Intra-oral examination during the first visit of the child patient at two years of age revealing the displaced 61 with only incisal edge clinically visible. (b) Intra-oral periapical radiograph revealing severe intrusive luxation injury in 61 as indicated by a red arrow line. (c) Post-operative image after six months following extraction of 61.

On clinical examination, intrusive luxation was diagnosed in tooth no. 61, with only the incisal third of the tooth being visible clinically (Figure 1a). Careful examination revealed a severe intrusion of 61 (7mm) when compared to the adjacent central incisor. An intraoral periapical radiograph revealed the possibility of impinging on the permanent tooth bud, which had its incisal third formed (Figure 1b). Hence, it was planned to extract the intruded tooth. The parents agreed, and written informed consent was obtained before extraction. After administering local anesthesia, tooth 61 was extracted, and the post-operative healing was uneventful (Figure 1c).

The parents were educated about the importance of periodic and meticulous follow-up until the permanent tooth eruption. An aesthetic functional space maintainer was not planned at this stage for the child owing to her age and potential behavior problems.

Follow-up and progress

**Figure 2:**
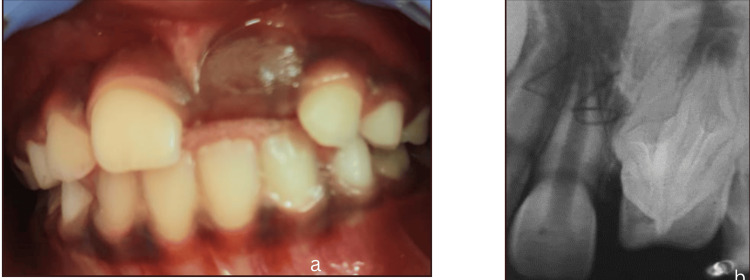
(a) Clinical photograph of the maxillary anterior region revealing unerupted 21 after eight years following trauma. (b) Intra-oral periapical radiograph showing gross malformation of crown and root of unerupted 21.

After periodic follow-ups for one year, the authors lost follow-up of the child due to relocation to a different city. After eight years, the parents reported back with the chief complaint of an unerupted upper front tooth. The chronological order of events following the intrusive luxation and the management of its complications with recalls are summarized in Table 1. Clinical examination revealed unerupted 21, but the teeth 11, 12, and 22 had erupted in place. Periapical radiograph revealed unerupted 21 with a gross malformation of the crown and the root (Figure 2b).

**Table 1:** Summary of events, examination, complications, and management of the intruded primary maxillary central incisor IOPA: intraoral periapical radiograph; CBCT: cone-beam computed tomography

Visit No	Follow-Up Time	Clinical Examination	Radiographic Examination	Management
1.	2-year-old child	Intrusion 61	7 mm intrusion	Extraction
2.	6 months	Completely healed soft tissues	-	-
3.	1 year follow-up	Dental caries: 74, restored	-	-
4.	8 years follow-up	Unerupted 21, erupted 11, 12, 22	IOPA showed gross malformation of 21. CBCT showed a combination of anomalies.	Wait and watch for tooth eruption
5.	8 years 6 months	Incisal one-third of the crown erupted in the oral cavity	-	-
6.	8 years 8 months	Periapical abscess in 21	-	Antibiotics and analgesics
7.	8 years 8 months 10 days	Healing of the periapical abscess	IOPA taken to study the canal morphology.	Endodontic treatment under the operating microscope.
8.	8 years 9 months	Asymptomatic. Complete healing of the soft tissues	Intact obturation	Light cure composite resin restoration, follow-up schedule
9	9 years 3 months	Asymptomatic	-	-

**Figure 3:**
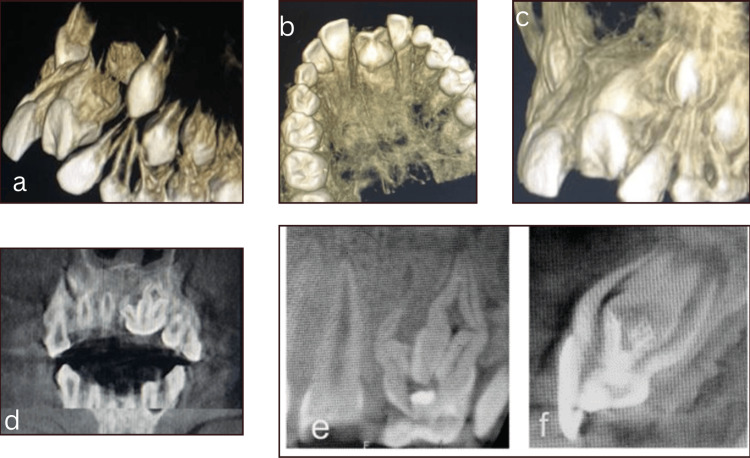
Cone beam CT images (a) Saggital 3D reconstructed view revealing the crown and root malformation in 21. (b) 3D reconstructed axial section view revealing the talon-cusp-like malformation in the incisal edge. (c) Saggital view showing crown and root dilaceration in 21. (d) Coronal sections revealing multiple roots in 21. (e) The complex morphology of the root is seen on the mesial surface. (f) The complex morphology of the root surface on the distal surface.

Cone-beam computed tomography (CBCT) images showed a combination of gross crown malformation with a talon cusp-like malformation on the palatal surface, root dilaceration, and multiple root formation (Figures 3a-3f).

**Figure 4:**
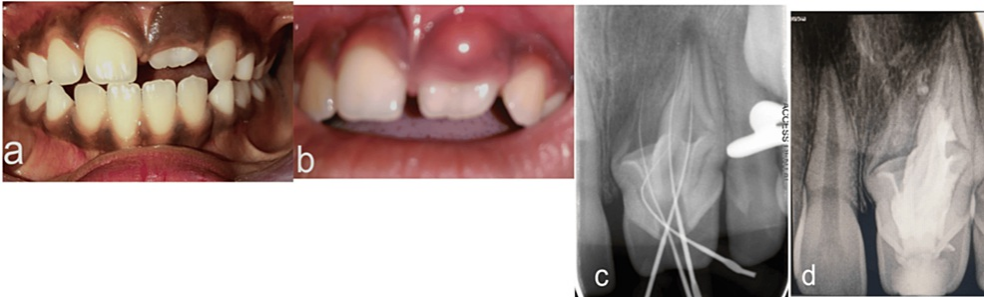
(a) Spontaneous clinical eruption of 21 noted after six months. (b) Periapical abscess noted after three months of clinical eruption in 21. (c) Intra-oral periapical radiograph revealing the complex morphology of root canal and negotiation of canals in 21. (d) Post-obturation radiograph of 21 following endodontic management under operative microscope.

Owing to the unusual morphology of the central incisor, the authors decided to wait and watch before any surgical intervention to facilitate eruption. Spontaneous eruption of 21 was noticed six months later (Figure 4a), with the incisal one-third of the tooth visible in the oral cavity.

During the next visit, the child developed an acute periapical abscess with respect to 21, and no previous history of trauma was reported on anamnesis (Figure 4b). Due to the complex anatomy of the crown and the root, endodontic treatment was carried out under the operating microscope. Multiple canals were located and negotiated during the treatment (Figure 4c). The canals were all thoroughly debrided, shaped, and finally obturated with gutta-percha (Figure 4d).

**Figure 5:**
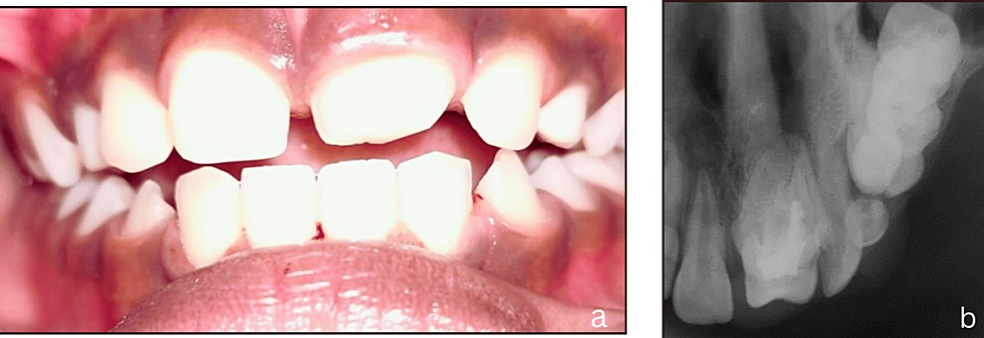
(a) Clinical photograph of 21 after six months of successful endodontic management and composite restoration. (b) Intra-oral periapical radiograph showing the same.

Esthetic modification of the crown was achieved using light cure composite resin restoration. The patient was asymptomatic at six months follow-up (Figures 5a-5b).

## Discussion

Trauma to the primary tooth causes changes in the development of permanent teeth ranging from 12% to 69% [6]. Several criteria must be addressed for the treatment of primary dentition trauma, including the patient's age, the developmental stage of the permanent successor at the time of damage, and the type of trauma to the primary tooth. The consequences range from yellowish-brown discoloration and/or developmental disruptions (hypo-calcification and/or hypoplasia) to a cessation of permanent bud development [10].

The present case report is a unique instance of sequelae in the permanent incisor caused by trauma to the predecessor, resulting in crown malformation (talon cusp-like appearance), root dilacerations, and duplication. The above findings have not yet been reported in the literature. In the current instance, the authors noticed such uncommon sequelae to the permanent central incisor following damage to the primary tooth at an age of less than three years. It was also discovered that the earlier trauma might have resulted in crown deformity and aberrant angulations of the root and multiple root canals in the upper left permanent central incisor at the age of 10 years. Chouchene et al. [11] observed similar findings who reported various anomalies in permanent teeth six years after an intrusive injury to the predecessors. Prabhakar et al. [12] noticed comparable anomalies in the root of the permanent successor as a result of trauma in the primary dentition. de Amorim et al. [13] observed that avulsion and invasive luxation were the most common types of trauma to the predecessor's teeth that resulted in dilacerations to their successor's teeth. This finding was congruent with the present clinical report, highlighting that severe intrusion could have been the etiological reason for such a mosaic of abnormalities.

Hattab [14] states that crown malformation occurs most commonly during the morpho-differentiation stage of tooth development as a result of the dental lamina's hyperproductivity. The inner enamel epithelium proliferates, resulting in out folding of the enamel and the creation of talon-like cusps [15]. The majority of permanent tooth dilacerations are caused by trauma to the preceding tooth, which displaces the already calcified part of the permanent germ while the rest of the tooth continues to grow at an aberrant angle [16]. According to Andreasen [6], such crown dilacerations occur in situations of injury between the ages of 1.5 and 3.5, while root deformity is the most common outcome between the ages of four and five. Altun [17] and Carvalho [18], on the other hand, found no relationship between the age of trauma to primary teeth and the type of long-term sequelae to permanent successors. These processes can be microscopic in nature, such as initiation, proliferation, histodifferentiation, morpho-differentiation, apposition, and maturation, or macroscopic in nature, such as initial calcification and different stages of crown and root formation (Nolla's staging) [6,9]. The above processes might be correlated with the present case report resulting in the permanent successor exhibiting severe crown deformity and several root dilacerations, resulting in a periapical infection that necessitated microscopic endodontic treatment.

The authors are unaware of any published reports on three types of malformations involving both crown and root in the same permanent tooth following primary tooth trauma: crown malformation, root dilaceration, and root multiplication.

This case underscores the intricate and often unpredictable nature of intrusive luxation injuries to primary teeth and their potential long-term implications on the developing permanent dentition. It emphasizes the importance of vigilant observation, timely intervention, and collaborative care in achieving favorable outcomes for patients with such TDI as observed in this case.

Clinical recommendations

The guidelines established by the International Association of Dental Traumatology (IADT) delineate the necessity of annual monitoring for a minimum of five years subsequent to the occurrence of trauma up to the emergence of the permanent tooth. The authors advocate for a multidisciplinary strategy that engages pediatric dentists, orthodontists, endodontists, and oral surgeons to effectively manage both immediate and delayed complications in instances of severe intrusion injuries as observed in our instance. Furthermore, the authors suggest periodic radiographic monitoring of the successor teeth post-eruption, drawing from the observations in this case after an eight-year follow-up period.

## Conclusions

The authors propose the establishment of a nationwide trauma registry specifically for dental injuries, aimed at overseeing the progression and complexities of these TDI. This registry is envisaged to yield extensive data spanning various regions of the nation, consequently enriching the protocols for treatment worldwide.

Moreover, the case report emphasizes the opportunity for discovering and confirming biomarkers that can anticipate the outcomes of TDI and the forecast of such TDI in primary dentition. The utilization of a chairside biomarker kit for the detection of TDI complications will be the focal point of investigation aimed at recognizing and managing through a multidisciplinary strategy.
